# No Integration Without Preparation: Building Workforce Capacity for Partner Services and HIV Molecular Epidemiology

**DOI:** 10.1093/ofid/ofaf761

**Published:** 2026-02-03

**Authors:** Naina Murthy, Carlos S Saldana

**Affiliations:** Division of Infectious Diseases, Department of Medicine, Emory University School of Medicine, Atlanta, Georgia, USA; Division of Infectious Diseases, Department of Medicine, Emory University School of Medicine, Atlanta, Georgia, USA

**Keywords:** HIV, molecular epidemiology, partner services

Integrating statewide partner services, confidential public health activities that identify, notify, test, and link partners of individuals diagnosed with HIV, with HIV molecular epidemiology (HME) holds significant potential to enhance HIV prevention. A recent study by Khanna et al [1] demonstrates this value linking Rhode Island's Contact Tracing Database (CTDB) with the HIV-1 Genomic Database (GDB). The authors found low overlap between the two sources. Importantly, 27% of named partners in the CTDB who underwent testing were newly diagnosed with HIV. This finding underscores the critical contribution of partner services. At the same time, the GDB revealed transmission links not captured through contact tracing alone, demonstrating its ability to uncover hidden connections and inform tailored public health interventions. The authors illustrate the complementary value of both approaches and the promise of combining them to generate a clearer view of the epidemic and guide HIV prevention and care [1].

Despite the encouraging case-finding yield of partner services and the additional transmission links revealed through HME, the capacity to translate these integrated approaches into routine practice remains limited across much of the USA [2]. Since 2018, when CDC required jurisdictions receiving HIV funding to implement HME for cluster detection and response [3], many health departments have struggled to operationalize these expectations in the face of strained infrastructure and workforce shortages [2]. Contact tracing staff already contend with longstanding challenges—stigma and mistrust, confidentiality concerns, declining engagement over repeated encounters, and low provider uptake [2–6]—and few are equipped with the tools and training needed to adapt these activities to the rapid transmission dynamics revealed by HME. Most staff conducting partner services interviews continue to rely on CDC's Passport to Partner Services [7], a curriculum developed in 2008 that standardizes interviewing and linkage practices but provides no guidance on molecular epidemiology. The long-anticipated replacement, *Principles, Practices, and Pathways to Disease Intervention*, has been delayed, leaving staff without updated direction and often improvising approaches ad hoc [8]. While national initiatives such as NASTAD's Integrated Learning Collaborative have begun to build shared knowledge and peer support, progress has been slow and inconsistent [9].

Importantly, HME does not replace traditional Partner Services but changes how they are conducted. Molecular cluster data can identify networks where transmission is accelerating and where interviews alone have not revealed the full extent of connections. This enables jurisdictions to prioritize interviews, re-engagement efforts, and field services in areas of highest need; escalate outreach in acute or recent infections; and tailor prevention and linkage activities to densely connected networks. These shifts require staff to integrate genetic cluster information into real-time decision-making and to communicate these concepts clearly and sensitively to patients, skills not covered in existing Partner Services curricula.

Our own data underscore these gaps [10]. Between November 2023 and December 2024, we conducted 18 focus group discussions with 92 participants, including 16 public health staff (partner services staff, HIV surveillance leads, and STI program managers) and 76 community members from priority populations (Black and Latino MSM, Black cisgender women, and Latina transgender women). Awareness of HME was uneven: while 87% of public health staff were familiar with the concept, only 37.5% had received HME-specific training, with some reporting they had “learned on the fly.” Community awareness also varied widely, ranging from 87.5% among Black MSM to 56.5% among Black cisgender women, with two-thirds of Latina transgender women and 62.5% of Latino MSM reporting familiarity. Community participants viewed HME with cautious optimism—recognizing its potential to improve prevention while expressing concerns about privacy, stigma, and the possibility of its misuse for HIV criminalization. Both public health staff and community members emphasized the need for ongoing, hands-on training that combines HME fundamentals with applied skills such as mock patient interviews, field visits, motivational interviewing, and syndemic frameworks, delivered through trauma-informed and culturally congruent approaches. Equally important were competencies in data privacy and protection, stigma reduction, and clear lay communication of HME to the public.

Taken together, this body of evidence points to an urgent need for investment in the workforce and infrastructure required to integrate partner services and HME effectively. Federal and state agencies must prioritize modernized, HME-specific training programs, updated curricula, and clear implementation guidance that reflect the realities of today's epidemic. Community engagement must remain central, as concerns around privacy, stigma, and trust will determine whether these tools are accepted and effective [11]. Additionally, academic–public health collaborations are essential for building the analytic, training, and implementation capacity needed to integrate partner services with HME. As highlighted by Khanna et al, such partnerships can support jurisdictions with data interpretation, workforce development, and community engagement. Recent work on HME community engagement further underscores the importance of trusted academic–community–public health relationships in advancing these approaches [12, 13]. Finally, integration efforts must be paired with broader policy reforms, including the abolition of HIV criminalization laws, which perpetuate stigma, erode trust, and undermine the very public health goals these strategies are designed to advance.

Without these steps, the complementary value shown in Rhode Island and reflected in our focus groups will remain an aspiration rather than a meaningful transformation. Aligning cutting-edge science, public health practice, policy, and community priorities is essential to ensure that molecular epidemiology strengthens the national HIV response.
